# The C_50_ carotenoid bacterioruberin regulates membrane fluidity in pink-pigmented *Arthrobacter* species

**DOI:** 10.1007/s00203-021-02719-3

**Published:** 2021-12-24

**Authors:** Alexander Flegler, André Lipski

**Affiliations:** Institute of Nutritional and Food Science, Food Microbiology and Hygiene, University of Bonn, Friedrich-Hirzebruch-Allee 7, 53115 Bonn, Germany

**Keywords:** *Arthrobacter*, Bacterioruberin, Carotenoid, Membrane fluidity, Cold adaptation

## Abstract

Carotenoids have several crucial biological functions and are part of the cold adaptation mechanism of some bacteria. Some pink-pigmented *Arthrobacter* species produce the rare C_50_ carotenoid bacterioruberin, whose function in these bacteria is unclear and is found mainly in halophilic archaea. Strains *Arthrobacter agilis* DSM 20550^T^ and *Arthrobacter bussei* DSM 109896^T^ show an increased bacterioruberin content if growth temperature is reduced from 30 down to 10 °C. In vivo anisotropy measurements with trimethylammonium-diphenylhexatriene showed increased membrane fluidity and a broadening phase transition with increased bacterioruberin content in the membrane at low-temperature growth. Suppression of bacterioruberin synthesis at 10 °C using sodium chloride confirmed the function of bacterioruberin in modulating membrane fluidity. Increased bacterioruberin content also correlated with increased cell resistance to freeze–thaw stress. These findings confirmed the adaptive function of bacterioruberin for growth at low temperatures for pink-pigmented *Arthrobacter* species.

## Main

To date, 1204 carotenoids of 722 source organisms have been identified and classified as C_30_, C_40,_ and C_50_ carotenoids depending on the number of carbons in their carotene backbones (Yabuzaki 2017, 2020). They are involved as accessory pigments in photosynthesis (Holt et al. 2005), act as antioxidants (Mandelli et al. 2012; Miller et al. 1996), light protection pigments (Shahmohammadi et al. 1998), oxidative stress protection (Giani and Martínez-Espinosa 2020), and membrane stabilizers (Lazrak et al. 1987). As lipophilic compounds, carotenoids are located in the cellular membrane, but their orientation within the membrane can vary depending on their chemical structure and the thickness of the membrane (Gruszecki 2004; Milon et al. 1986). More than 95% of all natural carotenoids are based on the symmetric C_40_ phytoene backbone, and only a small number of C_30_ and even fewer C_50_ carotenoids have been discovered (Tobias and Arnold 2006). Previous studies showed that carotenoids were able to lower the phase transition temperature of synthetic lipids (Subczynski et al. 1992, 1993), and this effect was dependent on the concentration of the pigment (Chaturvedi and Ramakrishna Kurup 1986; Strzałka and Gruszecki 1994; Subczynski et al. 1992). In accord with these observations, several authors argued that carotenoids might have a similar function in regulating membrane fluidity as sterols such as cholesterol or ergosterol in eukaryotic cells (Rohmer et al. 1979; Subczynski et al. 1992). Concerning the functions mentioned above, the involvement of carotenoids in bacterial cold adaptation was suspected especially for *Arthrobacter agilis*, *Micrococcus roseus*, and confirmed for *Staphylococcus xylosus* (Chattopadhyay et al. 1997; Fong et al. 2001; Seel et al. 2020; Strand et al. 1997).

The genus *Arthrobacter*, described by Conn and Dimmick (1947) and with an amendment by Busse (2016), is a predominant group of bacteria isolated from various sources such as soil, air, food, water, and plants, which has been found to produce a great variety of pigment hues, e.g., yellow, red, green, and blue (Sutthiwong et al. 2014). The species *A*. *agilis* and *Arthrobacter bussei* are known for the temperature-dependent pigmentation of the rare C_50_ carotenoid bacterioruberin and its glycosylated derivatives (Flegler et al. 2020; Fong et al. 2001)*.* Fong et al. (2001) suspected that bacterioruberin is involved in adapting *A*. *agilis* strain MB813 to low-temperature growth conditions, which was isolated from Antarctic sea ice by Bowman et al. (1997). Therefore, we assumed that bacterioruberin in pink-pigmented *Arthrobacter* species has a similar function in regulating membrane fluidity as the carotenoid staphyloxanthin in *S*. *xylosus*, a species that also shows intense pigmentation at 10 °C growth temperature but no pigmentation at 30 °C (Seel et al. 2020). This assumption is strengthened by the fact that, to date, all pink-pigmented *Arthrobacter* species have been isolated from low-temperature environments. Therefore, we hypothesized that bacterioruberin improves membrane properties under low-temperature conditions in *A*. *agilis* and *A*. *bussei* as model organisms. Using the methods employed in this work, we were able to relate changes in bacterioruberin content to changes in membrane fluidity by measuring the anisotropy of entire cells. Moreover, freeze–thaw stress tests demonstrate that the carotenoids also stabilize the cell membrane.

Comparative fatty acid profiles have already been established for strains *A*. *agilis* DSM 20550^T^ and *A*. *bussei* DSM 109896^T^ at 10 and 30 °C growth temperature (Flegler et al. 2020). Both *Arthrobacter* species showed an adaptive response to low growth temperature, mainly based on the increase of unsaturated FAs for *A*. *agilis* and a shift from *iso*-branched to *anteiso*-branched fatty acid for *A*. *bussei*, respectively. The difference of the weighted average melting temperature (ΔWAMT) between cultures grown at 10 and 30 °C was calculated using the available percentage fatty acid abundance to derive the extent of FA-dependent temperature adaptation as described by Seel et al. (2020). In addition, we calculated ΔWAMT values from the fatty acid profiles published previously, based on the melting temperatures of free fatty acid given by Knothe and Dunn (2009). Both organisms showed a similar but moderate alteration in fatty acid composition resulting in a ΔWAMT of about 3 °C for *A*. *agilis* and 6.7 °C for *A*. *bussei*. Thus, FA-dependent cold adaptation was lower in *A*. *agilis* compared to *A*. *bussei*.

### Increased bacterioruberin content alter membrane fluidity and support cold adaptation

**Fig. 1:**
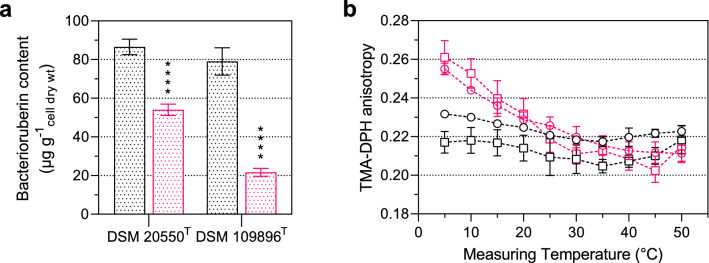
Temperature-dependent bacterioruberin content and membrane fluidity. **a** Total bacterioruberin content of strains *Arthrobacter agilis* DSM 20550^T^ and *Arthrobacter bussei* DSM 109896^T^ grown at 10 °C (black) or 30 °C (red). **b** Membrane fluidity analyzed by TMA-DPH anisotropy of strains *Arthrobacter agilis* DSM 20550^T^ (squares) and *Arthrobacter bussei* DSM 109896^T^ (circles) grown at 10 °C (black) and 30 °C (red). Values are means ± standard deviation (**a**: *n* = 6, **b**: *n* = 3). Asterisks represent *p* values (*p < 0.001, **p < 0.0001, ***p < 0.00001, ****p < 0.000001) compared to cultures grown at 10 °C (colour figure online)

According to previous observations, the colonies of both *Arthrobacter* strains showed more pronounced pigmentation at low-temperature growth. The total carotenoid content of bacterioruberin and its glycosylated derivates was extracted with high-performance liquid chromatography (HPLC) as described by Kaiser et al. (2007) and Seel et al. (2020) and quantified as β-carotene equivalents using an external calibration curve. The quantitative HPLC analysis confirmed a significant increase in bacterioruberin at a growth temperature of 10 °C by about 60.4% in *A*. *agilis* and 264.1% in *A*. *bussei*. The total bacterioruberin content was higher in *A*. *agilis* compared to *A*. *bussei* if both strains were grown at 30 °C (Fig. 1a). Anisotropy was measured to determine a correlation between the membrane fluidity and the bacterioruberin content. Sample preparation and membrane fluidity determination by trimethylammonium-diphenylhexatriene (TMA-DPH) anisotropy measurement were performed as Seel et al. (2018) described. The results showed a similar behavior of the two strains with an evident influence of the higher bacterioruberin content at low growth temperatures (Fig. 1b). Growth at 30 °C significantly reduced the bacterioruberin content of both *Arthrobacter* species, which showed an approximating phase transition, indicated by the changed curve of measured anisotropy in a temperature range of 5–50 °C. Characteristically, constant anisotropy values at the measurement range limits indicate the membranes’ complete phase transition from the gel-like solid-state (high anisotropy) to the liquid-crystalline fluid state (low anisotropy). Cultures grown at 10 °C with high bacterioruberin content showed no pronounced phase transitions in both species, evident from the slight increase of anisotropy with a decrease in temperature. However, *A*. *bussei* DSM 109896^T^ with the lower bacterioruberin content at 10 °C exhibited higher average anisotropy values than *A*. *agilis* strain DSM 20550^T^. Nevertheless, the membrane fluidity in both species was almost constant over the entire measured temperature range indicating a membrane fluidizing effect of the *Arthrobacter* carotenoids at low temperatures.

**Fig. 2:**
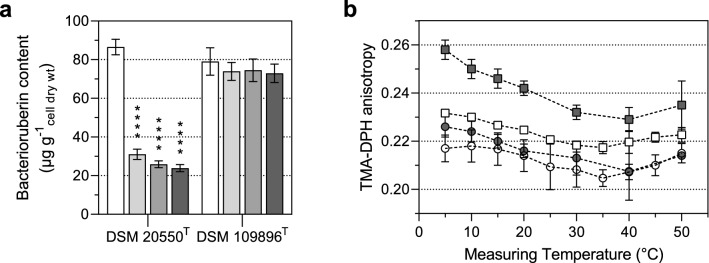
Sodium chloride (NaCl)-dependent bacterioruberin content and membrane fluidity. **a** Total bacterioruberin content of strains *Arthrobacter agilis* DSM 20550^T^ and *Arthrobacter bussei* DSM 109896^T^ grown at 10 °C in tryptic soy broth supplemented with 0% (white), 2% (light grey), 3% (grey) or 4% (dark grey) (wt/vol) NaCl. **(b)** Membrane fluidity analyzed by TMA-DPH anisotropy of strains *Arthrobacter agilis* DSM 20550^T^ (squares) and *Arthrobacter bussei* DSM 109896^T^ (circles) grown at 10 °C in tryptic soy broth without NaCl (white) or with 4% (wt/vol) NaCl (dark grey). Values are means ± standard deviation (*n* = 3). Asterisks represent *p* values (*p < 0.001, **p < 0.0001, ***p < 0.00001, ****p < 0.000001) compared to cultures grown at 10 °C

To confirm the effects of bacterioruberin content on membrane fluidity, we suppressed its synthesis for both *Arthrobacter* species by diphenylamine supplementation, according to Hammond and White (1970). Diphenylamine concentrations of 25, 50, and 75 µM significantly reduced the bacterioruberin content in both tested species grown at 10 °C (data not shown). This approach, however, proved to be unsuitable because diphenylamine itself significantly alters membrane properties due to its lipophilic character, as already mentioned by Seel et al. (2020). Alternatively, to verify the effect of bacterioruberin on membrane fluidity, we suppressed bacterioruberin synthesis by adding sodium chloride (NaCl), as achieved previously by Fong et al. (2001). A correlation between NaCl concentration and bacterioruberin synthesis was already reported for *A. agilis* and *S*. *xylosus*. We detected a similar correlation for *A*. *agilis* DSM 20550^T^ but not for *A*. *bussei* DSM 109896^T^ (Fig. 2a). Supplementation of 2, 3, or 4% (wt/vol) NaCl significantly reduced the bacterioruberin content of *A*. *agilis* by about 64.2%, 70.9%, or 72.4%, respectively. This allows verifying the effect of bacterioruberin on the biophysical parameters of the cell membrane without changing the cultivation temperature. The decreased bacterioruberin content of *A*. *agilis* showed a clear impact on membrane fluidity (Fig. 2b). The anisotropy values showed a similar progression of the phase transition pattern between *A*. *agilis* cells grown at 30 °C and cells supplemented with 4% NaCl at 10 °C. This resulted in a loss of membrane fluidity at low temperatures. On the other hand, *A*. *bussei* retained a higher membrane fluidity, which is related to the almost unchanged bacterioruberin content. These results confirmed that the measured effect on membrane fluidity was due to the cells’ bacterioruberin content.

### Bacterioruberin content affects resistance to temperature stress

**Fig. 3:**
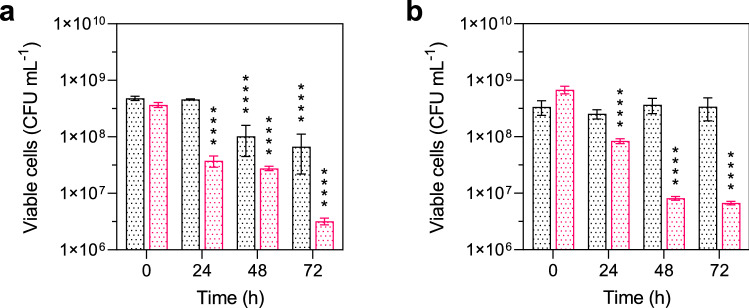
Freeze–thaw stress test. Viable cell count of strains **a** *Arthrobacter agilis* DSM 20550^T^ and **b** *Arthrobacter bussei* DSM 109896^T^ grown at 10 °C (black) and 30 °C (red) in tryptic soy broth after one (24 h), two (48 h) and three (72 h) freeze–thaw cycles. Values are means ± standard deviation (*n* = 3). Asterisks represent *p* values (*p < 0.001, **p < 0.0001, ***p < 0.00001, ****p < 0.000001) compared to the initial cell count at 0 h (colour figure online)

It is unknown if the thermotropic phase transition by higher amounts of bacterioruberin finally impacts bacterial cell fitness under low-temperature conditions. In this study, using the term fitness as a quantitative attribute for the survival of an external stressor, the fitness of bacterial cells was tested by exposing them to freeze–thaw stress. Freeze–thaw stress resistance is a recognized indicator of cell membrane integrity and bacterial cell fitness (Carlquist et al. 2012; Flegler et al. 2021; Sleight et al. 2006). To confirm the correlation between bacterioruberin content and cell fitness, we achieved a reduced bacterioruberin content in both strains by cultivation at 30 °C. The freeze–thaw stress test was performed according to Flegler et al. (2021). Indeed, freeze–thaw stress tests showed the positive effect of bacterioruberin on membrane integrity. *A*. *agilis* and *A*. *bussei* significantly reduced the number of viable cells grown at 30 °C compared to cultures grown at 10 °C (Fig. 3). Cells grown at 30 °C gradually decreased in the number of viable cells after each freeze–thaw cycle to a minimum of about 6.7 × 10^6^ and 3.2 × 10^6^ CFU mL^−1^ for *A*. *agilis* and *A*. *bussei*, respectively, after the third freeze–thaw step. Almost no reduction in viable cells was measured for *A*. *bussei* grown at 10 °C. In contrast, *A*. *agilis* grown at 10 °C showed a slight decrease of viable cells after the second freeze–thaw step. These results confirmed the beneficial effect of an elevated bacterioruberin content on cell membrane integrity at 10 °C in both species.

### Conclusion

This work reveals the beneficial effect of the bacterioruberin content of pink-pigmented *Arthrobacter* species on membrane fluidity under low-temperature conditions. These results strengthen that bacterioruberin is a fatty acid-independent mechanism for regulating membrane fluidity and represents an additional adaptive response to low growth temperatures with a beneficial impact on membrane integrity, as demonstrated by the increased resistance to freeze–thaw stress. The beneficial effect of this rare C_50_ carotenoid on cells of *Arthrobacter* species and other bacterioruberin-producing bacteria may in part explain the successful colonization of low-temperature environments by these organisms.

## Materials and methods

### Materials

All chemical reagents and solvents were purchased from Alfa Aesar, Carl Roth, MilliporeSigma, Sigma-Aldrich, Thermo Fisher Scientific, and VWR. Solvents and water for analytics were of HPLC grade and used as received.

### Bacterial strains, culture media, and cultivation

In this research, we examined two *Arthrobacter* strains. *A*. *agilis* DSM 20550^T^ was isolated in 1981, and *A*. *bussei* DSM 109896^T^ was isolated from cheese made of cow’s milk in 2018. Both species belong to the “Pink *Arthrobacter agilis* group” within the “*Arthrobacter agilis* group” and showed a more intense pigmentation at low growth temperatures (Flegler et al. 2020).

All species were aerobically cultured in 100 mL tryptic soy broth (TSB). TSB contained 17.0 g peptone from casein L^−1^, 3.0 g peptone from soy L^−1^, 2.5 g d-glucose L^−1^, 5.0 g sodium chloride L^−1^, and 2.5 g dipotassium hydrogen phosphate L^−1^ using 300 mL Erlenmeyer flasks. Growth in the TSB was documented by optical density (OD) at 625 nm with a GENESYS 30 visible spectrophotometer (Thermo Fisher Scientific, USA). Cultures were prepared in independent replicates, inoculated with 1% (vol/vol) of overnight culture, and incubated on an orbital shaker at 10 or 30 °C and 150 rpm in the dark until late exponential phase (OD_625nm_ = 1–1.2). Cultures were harvested by centrifugation (10,000×*g* for 10 min) at growth temperature and washed thrice with sterile phosphate-buffered saline (PBS), which was adjusted to growth temperature, pH 7.4. Subsequently, this biomass was used for carotenoid analysis, membrane fluidity measurement, and freeze–thaw stress test. Colonies were cultivated on tryptic soy agar (TSA) containing 15.0 g peptone from casein L^−1^, 5.0 g peptone from soy L^−1^, 5.0 g sodium chloride L^−1^, and 15.0 g agar–agar L^−1^ at 30 °C.

To determine colony-forming units (CFU) for the freeze–thaw stress test, serial dilutions were plated on TSA (90 mm Petri dish) using the exponential mode (ISO 4833-2, ISO 7218, and AOAC 977.27) of the easySpiral automatic plater (Interscience, France). After incubation for 48 h at 30 °C, the CFU were counted for the corresponding dilution steps, and the weighted average of enumerated *Arthrobacter* sp*.* was given in CFU mL^−1^. The results for the temperature stress test were presented as viable cells (CFU mL^−1^).

### Statistical evaluation

Statistical analysis was performed using Prism (version 9.2.0; GraphPad Software, United States). Mean values (*M*) and standard deviations (SD) of *n* (see legends) biological replicates were calculated for all experiments. Two-way ANOVA was performed with the recommended post hoc test (*α* = 0.001). Data are presented as *M* ± SD; *p < 0.001, **p < 0.0001, ***p < 0.00001, ****p < 0.000001.

## Declarations

### Data availability

The data sets generated and analyzed during the current study are available on request from the corresponding author.
